# Dynamic in vitro culture of bovine and human ovarian tissue enhances follicle progression and health

**DOI:** 10.1038/s41598-023-37086-0

**Published:** 2023-07-21

**Authors:** Vincenza Barbato, Vincenzo Genovese, Vincenza De Gregorio, Maddalena Di Nardo, Angela Travaglione, Luigi De Napoli, Gionata Fragomeni, Elisabetta M. Zanetti, Satish K. Adiga, Giuseppe Mondrone, Thomas D’Hooghe, Wengijng Zheng, Salvatore Longobardi, Gerardo Catapano, Roberto Gualtieri, Riccardo Talevi

**Affiliations:** 1 Department of Biology, University of Naples “Federico II”, Complesso Universitario Di Monte S. Angelo, Via Cinthia, 80126 Naples, Italy; 2 Department of Mechanical, Energy and Management Engineering, University of Calabria, Via P. Bucci, 87030 Rende, CS Italy; 3 Department of Medical and Surgical Sciences, Magna Graecia University, Viale Europa - Loc. Germaneto, 88100 Catanzaro, Italy; 4 Department of Engineering, University of Perugia, 06125 Perugia, Italy; 5 Centre of Excellence in Clinical Embryology, Department of Reproductive Science, Kasturba Medical College, Manipal Academy of Higher Education, Manipal, 576 104 India; 6 IVF Research, Education, Development S.R.L., Via Josemaria Escrivà, 68, 81100 Caserta, Italy; 7 Global Medical Unit Fertility, Merck Healthcare KGaA, Frankfurter Strasse 250, 64293 Darmstadt, Germany; 8 Department of Development and Regeneration, Group Biomedical Sciences, KU Leuven (Leuven University), Gasthuisberg Campus, Herestraat 49, 3000 Leuven, Belgium; 9 Present Address: Institute for Biomedical Technologies ITB, National Research Council CNR, Via Moruzzi, 1, 56124 Pisa, Italy

**Keywords:** Biological techniques, Biotechnology

## Abstract

In vitro ovarian cortical tissue culture, followed by culture of isolated secondary follicles, is a promising future option for production of mature oocytes. Although efforts have been made to improve the culture outcome by changing the medium composition, so far, most studies used static culture systems. Here we describe the outcome of 7 days cultures of bovine and human ovarian cortical tissue in a dynamic system using a novel perifusion bioreactor in comparison to static culture in conventional and/or gas permeable dishes. Findings show that dynamic culture significantly improves follicle quality and viability, percentage and health of secondary follicles, overall tissue health, and steroid secretion in both species. Model predictions suggest that such amelioration can be mediated by an enhanced oxygen availability and/or by fluid-mechanical shear stresses and solid compressive strains exerted on the tissue.

## Introduction

One of the most ambitious and challenging topics in reproductive biology is the exploitation of the huge reserve of dormant primordial ovarian follicles to produce mature and developmentally competent oocytes by replicating in vitro the natural folliculogenesis. The possibility to generate a significant number of mature oocytes from small ovarian cortical slices cultured in vitro would revolutionize current animal breeding procedures, preservation of endangered species, establishment of oocyte banks, and management of fertility preservation and in vitro fertilization (IVF) procedures in human assisted reproduction. Although such a goal has been achieved in mice more than twenty years ago^1,2^, the initial enthusiasm of scientists collided with the enormous difficulties in replicating these procedures in large mammals and humans^3,4^. Several anatomical and functional differences hinder the translation of the mouse results to large mammals and humans, such as the duration of folliculogenesis (about 20 days in mice vs more than 180 days in humans and cattle)^5–7^, the age of the biological starting material (newborn ovaries in mice vs adult ovaries in humans), the size of the developing follicles, the mechanical consistency of the ovarian tissue, to quote but a few. Recently, two human studies^8,9^ have rekindled the interest in this field and have demonstrated that some mature oocytes, albeit with limited yield and normality, can be generated in vitro from dormant primordial follicles using a multistep culture system. The first step of this in vitro folliculogenesis consists of the organ culture of cortical ovarian tissue slices, in which dormant follicles are activated and progress through the primary and early secondary stage. Little is known about primordial follicles activation and preantral growth despite the several investigations performed to elucidate the crucial factors involved in antral and preovulatory follicles development^10–12^. In the human, this phase of initial follicle development in vivo lasts approximately 120 days and depends on a complex and incompletely elucidated interplay among the oocyte, the granulosa and stromal cells, and the extracellular matrix^3,7,13^. After unsuccessful attempts to grow isolated primordial follicles^14,15^, a consensus has been reached that the development of primordial follicles to the secondary stage can only be achieved by culturing the intact ovarian cortex^16^.

We recently demonstrated that oxygen availability may play a key role during culture of bovine and human ovarian cortical tissue (BOCT and HOCT, respectively)^17^. In particular, we showed that in the static culture of BOCT and HOCT strips in dishes with gas-permeable bottom (PD), in which oxygen is supplied to tissue from both the top and the bottom of the dish, follicle health and progression is enhanced by an optimal range of oxygen availability in situ, as predicted by a transport model of oxygen from the medium bulk into the tissue strips under varying culture conditions. Culturing ovarian tissue in bioreactors ensuring an adequate oxygen supply may be expected to contribute to better follicle health and growth. Another important limitation to current static ovarian culture systems is the deprivation of nutrients and the accumulation of waste products in the stagnant medium layer adjacent to tissue. A dynamic culture system would provide a continuous flow of culture medium towards and around tissue, which could promote the continuous exchange of nutrients and metabolites from medium to tissue and vice-versa. This might help overcome such limitations and better mimic in vitro the physiological processes of early folliculogenesis. Dynamic bioreactors in which medium perfuses cells seeded in porous constructs enabling control and monitoring of operating parameters that affect cell culture efficiency, and maintaining long-term cell viability have been successfully developed for some organs/tissues but not for ovarian tissue^18–21^. In fact, ovarian tissue perfusion is made impossible by its very low hydraulic permeability^22–24^. Our group has recently designed and developed a dynamic bioreactor that enhances the exchange of oxygen and nutrients between medium and tissue by flowing medium around the tissue strips in suitable patterns (i.e., in perifusion mode). At the same time, tissue is subjected to steady mechanical stimulation, in terms of uniaxial solid compressive strain and fluid-mechanical shear stresses^25^.

Herein, we report the characterization of the performance of such a novel dynamic bioreactor for the extended perifusion culture of BOCT and HOCT strips. Performance of the perifusion bioreactor (PB) was compared to static in vitro culture in dishes with either conventional gas-impermeable (CD) or gas-permeable (PD) bottom. The main results showed that culture of both BOCT and HOCT strips in the perifusion bioreactor improves the number and quality of secondary follicles, follicle viability and hormone secretion, and the viability of tissue as a whole, when compared to either static culture system.

## Results

### Model predictions for static and dynamic culture

**Figure 1:**
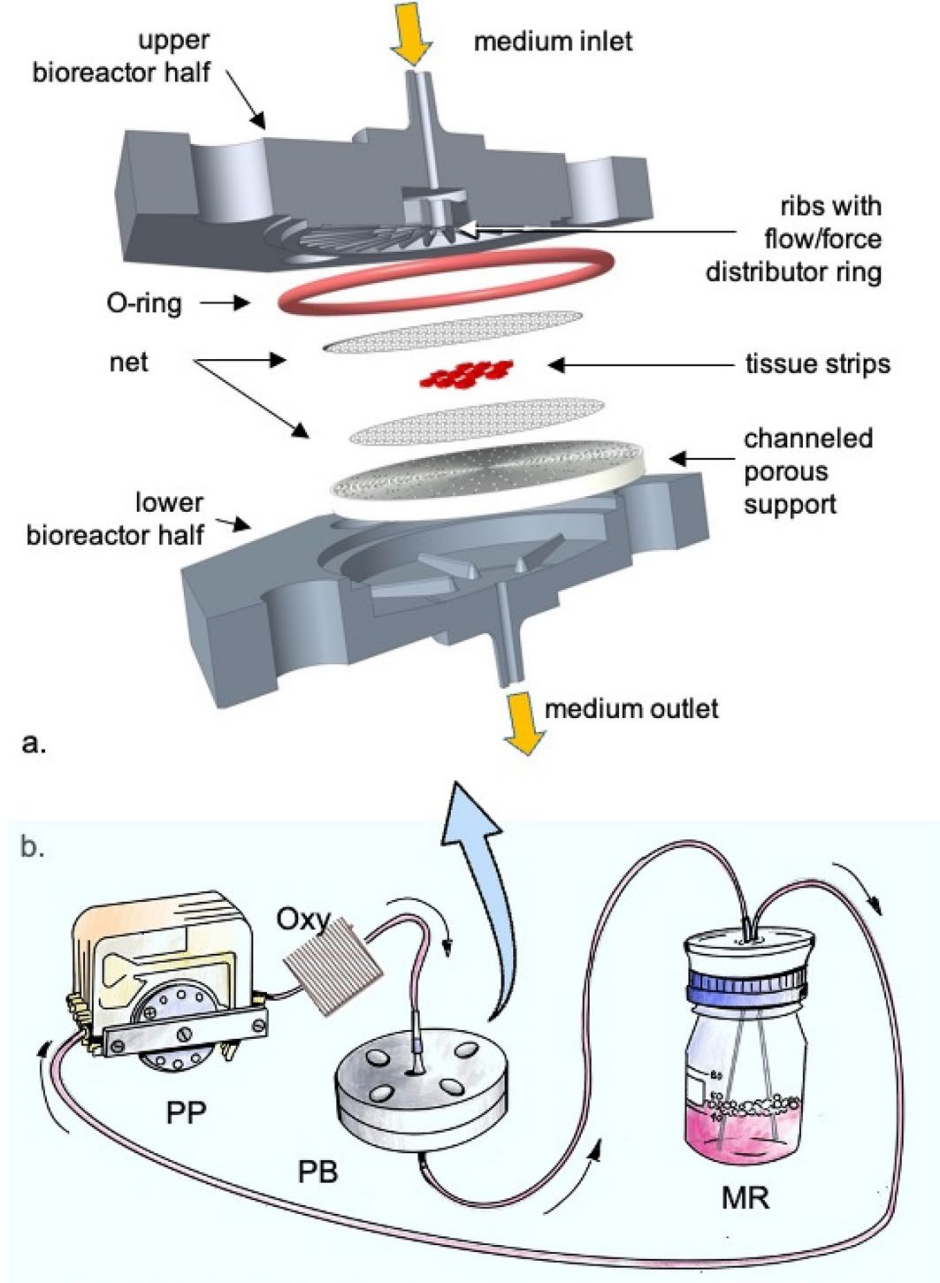
Scheme of bioreactor and experimental set-up. Exploded view of the perifusion bioreactor components (**a**) and their assembly (**b**); drawing of the experimental set-up for tissue culture: MR—medium reservoir; Oxy—medium oxygenator; PB—perifusion bioreactor; PP—peristaltic pump.

**Figure 2:**
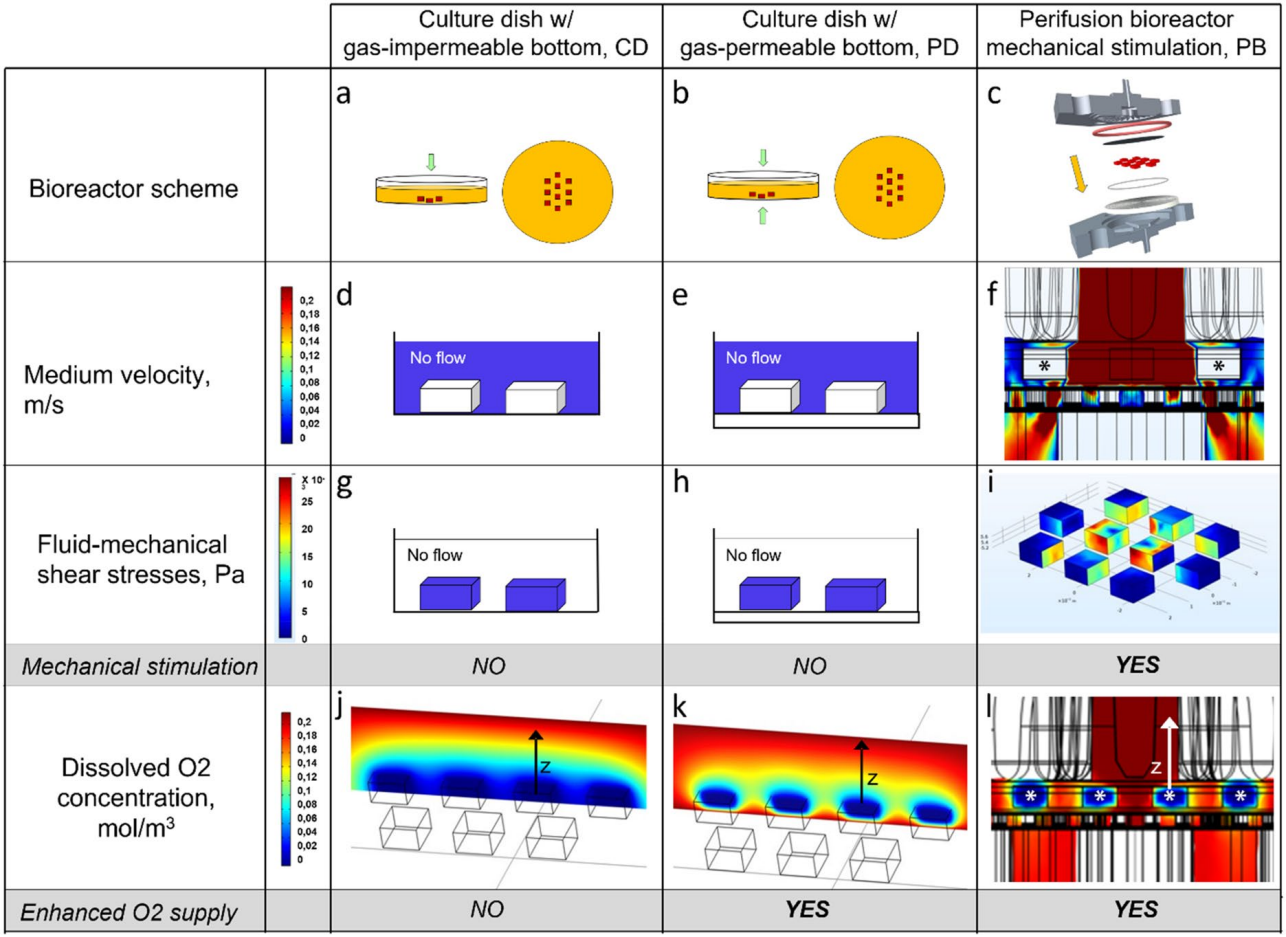
Flow and oxygen transport model predictions. (**a-c**) Bioreactor scheme: (**a**) static conventional dish (CD), (**b**) gas-permeable dish (PD), and **(c**) dynamic perifusion (PB) bioreactors; (**d**-**f**) Medium velocity contours at middle plane of various bioreactors: (**d**) CD; (**e**) PD; and (**f**) PB; (**g–i**) Fluid-mechanical shear stress at middle plane of various bioreactors: (**g**) CD; (**h**) PD; and (**i**) PB; (**j–l**) Dissolved oxygen concentration at middle plane of various bioreactors: (**j**) CD; (**k**) PD; and (**l**) PB. In figures (**j–l**), the z-axis is reported to indicate the direction along which the dissolved oxygen concentration profiles develop upstream from the tissue strip. Operating conditions and parameter values reported in text. *position of the tissue strips.

The scheme of the novel dynamic bioreactor, the culture dishes and the experimental set-up used for this study are shown in Figs. 1a, b and 2a–c. The predictions of the flow transport model^26^ show that the PB allows perifusion with medium of all the outer surfaces of the tissue strips thereby permitting an effective exchange of solutes between tissue and medium, as Fig. 2d–f show. Figure 2g–i show that the flow transport model predicts that during culture in PB the tissue strips are also subjected to fluid-mechanical shear stresses, of higher intensity on strips located at the center of the culture chamber and of decreasing intensity towards the periphery, that together with the applied solid compressive strains, are expected to contribute to eliciting tissue biomechanical response. Consistent with the flow transport model predictions, the oxygen transport model predicts that the effective perifusion of tissue with medium contributes to increasing the average dissolved oxygen concentration around and inside the strips as compared to CD and PD culture (Fig. 2j–l).

### Bovine ovarian cortical tissue culture

**Figure 3:**
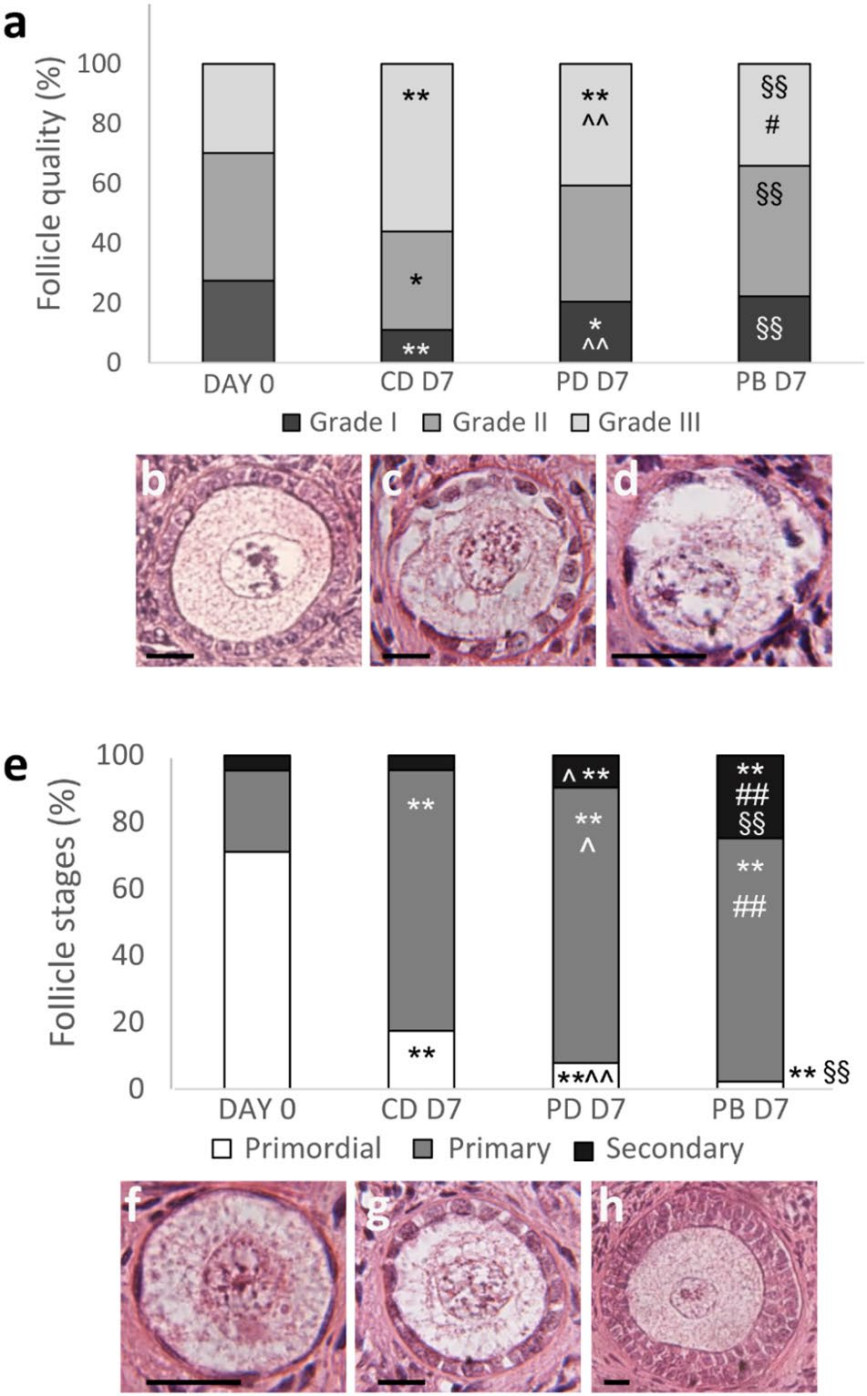
Histological characterization of fresh and cultured BOCT. (**a**) Follicle quality in BOCT cultured under static (CD and PD) and dynamic (PB) conditions versus fresh tissue (D0). (**b-d**) Representative images of grade I (**b**), II (**c**) and III (**d**) follicles. (**e**) Follicle stages in BOCT cultured in static (CD and PD) and dynamic (PB) conditions versus fresh tissue (D0); (**f–h**) Representative images of primordial (**f**), primary (**g**) and secondary (**h**) follicles. Bar = 20 μm. All the experiments were performed in triplicate (n = 3), and the values represent cumulative percentages. *P < 0.05 and **P < 0.01 versus D0; #P < 0.05 and ##P < 0.01 PB versus PD; §§P < 0.01 PB versus CD; ^P < 0.05 and ^^P < 0.01 PD versus CD.

**Figure 4:**
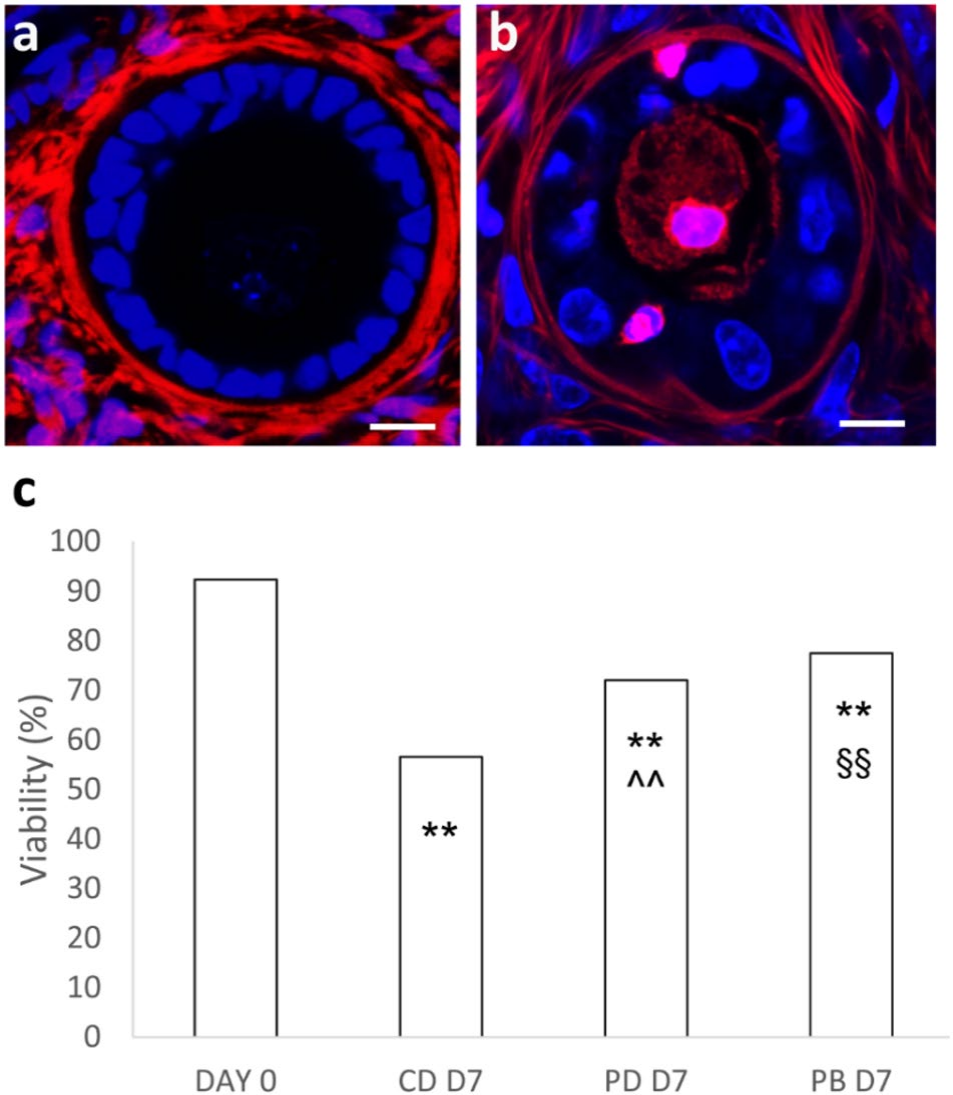
Follicle viability in fresh and cultured BOCT. (**a**) Follicle viability of BOCT cultured under static (CD and PD) and dynamic conditions (PB) versus fresh tissue (D0). (**b**, **c**) Representative confocal micrographs of a live (**b**), and a dead (**c**) follicle. Blue, Hoechst 33342 ± stained nuclei; red, extracellular matrix and dead cells stained by live ± dead far-red; magenta, nuclei of dead cells. Bar = 20 μm. All the experiments were performed in triplicate (n = 3), and the values represent cumulative percentages. **P < 0.01 versus D0; §§P < 0.01 PB versus CD; ^^P < 0.01 PD versus CD.

Follicle quality and stages (Fig. 3a and b, respectively) of BOCT strips were assessed after 7 days of culture by histological characterization of tissue in dynamic PB and in static (i.e., CD and PD) culture, and were compared with fresh tissue harvested at D0 (n = 3; total follicle number, 1875: D0—545; D7—606, CD; 259, PD; 465, PB). Interestingly, BOCT cultured in PB exhibited a proportion of healthy follicles (grade I and II) and atretic follicles (grade III) comparable with fresh tissue D0. The proportion of atretic follicles (grade III) in strips cultured for 7 days in CD and PD was significantly higher than in fresh tissue (i.e., D0) (Fig. 3a). The histological examination revealed that the strips cultured in PB and PD harboured more grade I follicles than in CD at the end of culture (i.e., D7). The strips cultured in PB and PD at D7 exhibited a noticeable and statistically significant (P < 0.01) decrease in primordial follicles and a concurrently substantial increase in primary and secondary follicles when compared to fresh tissue at D0 (Fig. 3b). Culture for 7 days in PB enhanced follicle progression, yielding a higher percentage of secondary follicles even than culture in PD. A smaller percentage of follicles progressed to the secondary stage in strips cultured in static CD than in both the dynamic PB and the static PD. Assessment of follicle viability at the confocal microscope (n = 3; total follicle number, 1650: D0, 409; D7—424, CD; 396, PD; 421, PB) allows the identification of viable (Fig. 4a) and dead (Fig. 4b) follicles. A significant decrease of viability was observed in all cultured samples as compared to fresh tissue (D0, 92.4%). However, at D7 follicle viability in PB (77.5%) and PD (72.1%) was significantly higher than in tissue cultured in parallel in CD (56.6%) (Fig. 4c).

**Figure 5:**
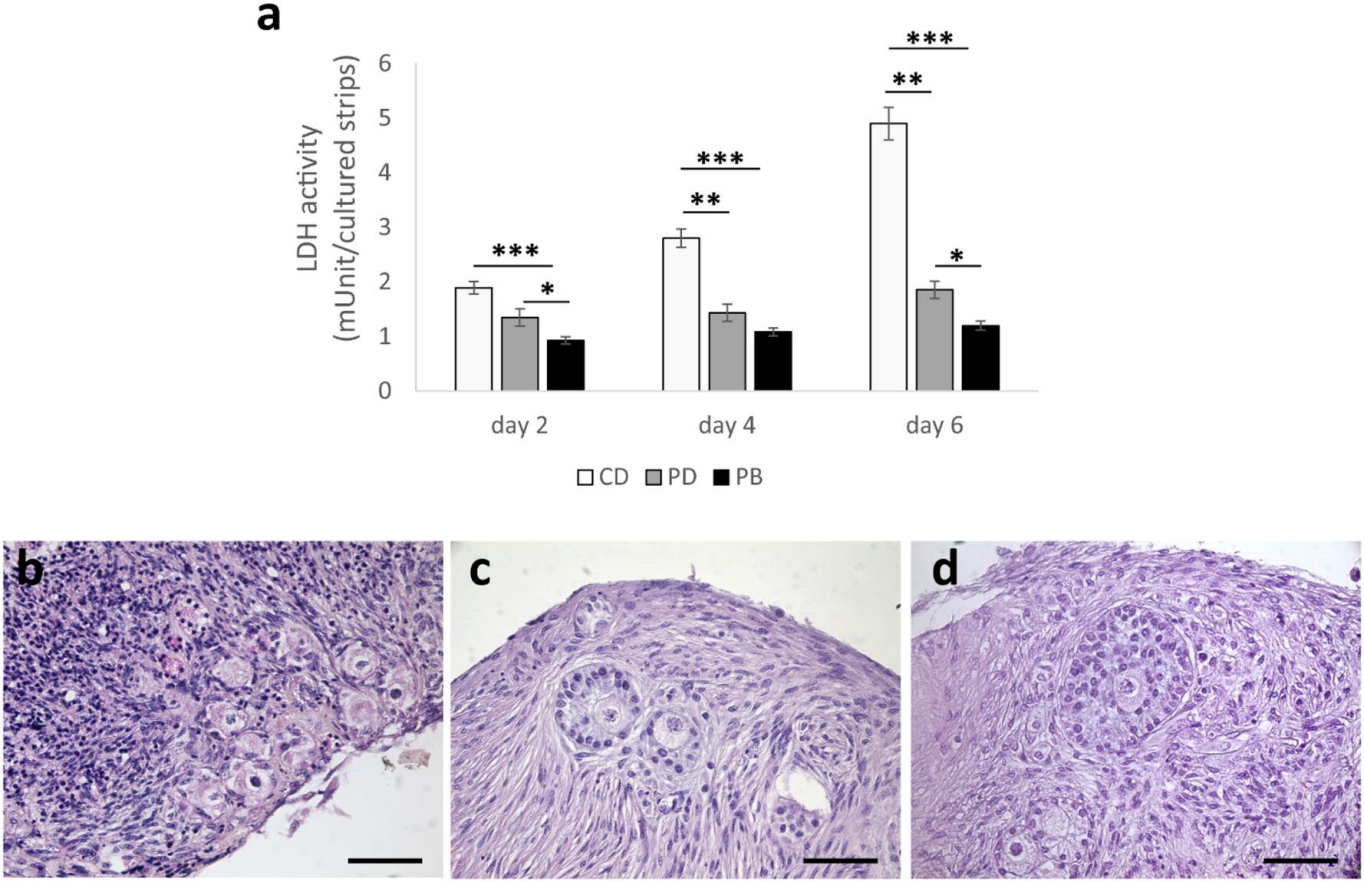
Evaluation of whole tissue viability during BOCT culture under different conditions. (**a**) LDH activity (mUnits/10 cultured strips) of BOCT cultured under static (CD and PD) and dynamic (PB) conditions. All the experiments were performed in triplicate (n = 3), data are presented as mean ± standard deviation; *P < 0.05, **P < 0.01 ***P < 0.001. (**b-d**) Representative images of BOCT cultured in static (CD) (**b**), (PD) (**c**), and dynamic (PB) (**d**) conditions after 7 days of culture; Bar = 50 μm.

Measurement of the activity of cytoplasmic enzymes released by damaged cells is a valuable method for cell death assessment. To this end, Lactate Dehydrogenase (LDH) activity was measured in the culture medium to highlight both follicles and stromal cells viability. In our study, LDH was estimated every other day in the spent medium collected from PB, PD and CD cultures to assess the overall tissue viability. Remarkably, a lower level of LDH was detected in the media of strips in dynamic PB than in static CD and PD culture. LDH level was significantly lower (P < 0.001) at all time points in PB than in CD, and at D2 and D6 in PB than in PD (Fig. 5a). Histological analysis are in agreement with these results. In particular, BOCT strips cultured for 7 days in PB (Fig. 5d) and PD (Fig. 5c) exhibited a regular distribution of healthy stromal and granulosa cells around an intact oocyte. They also exhibited a lower number of pyknotic stromal cells and atretic follicles in advanced phases of reabsorption than in CD (Fig. 5b).

### Human ovarian cortical tissue culture

**Figure 6:**
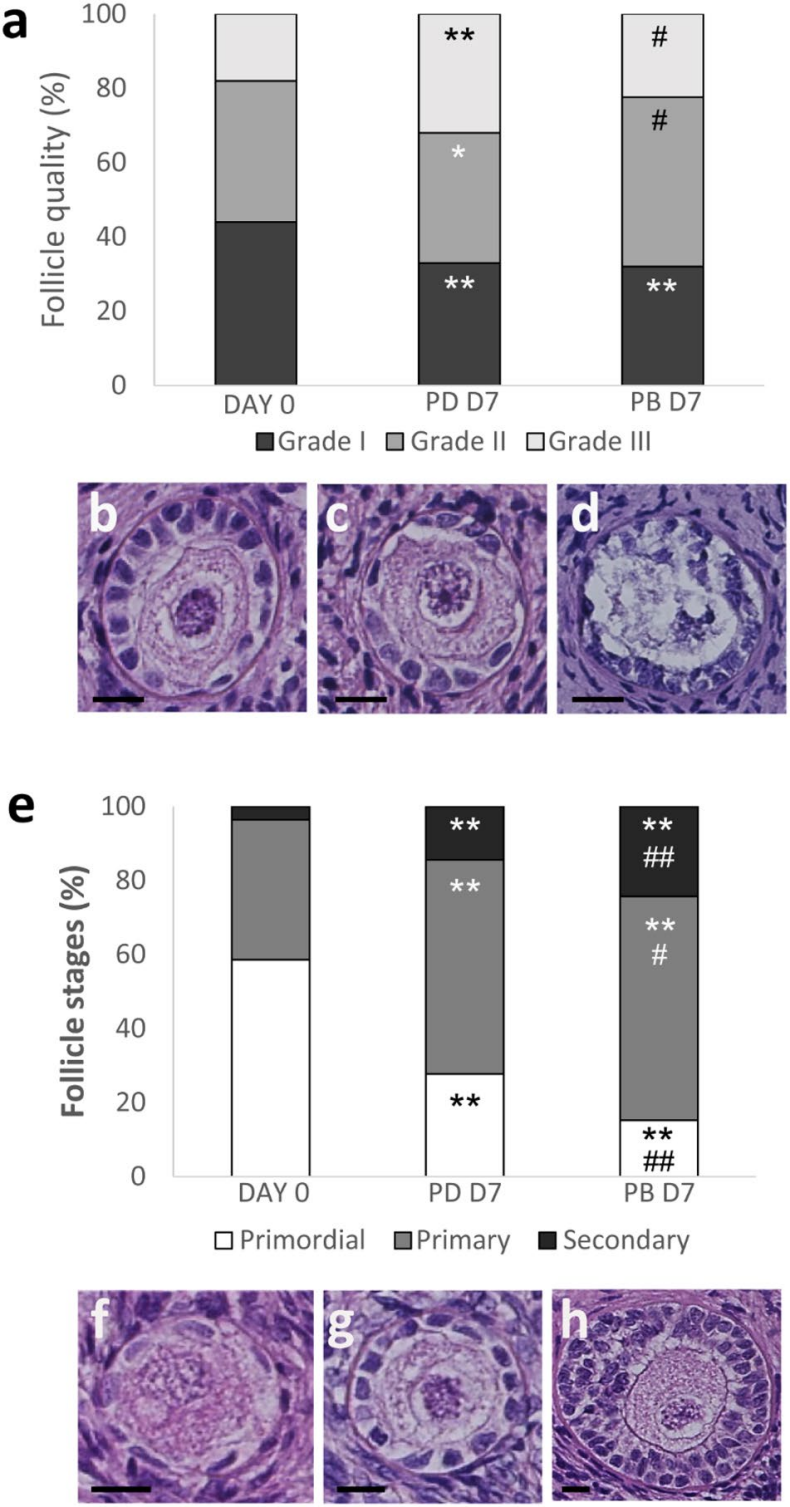
Histological characterization of fresh and cultured HOCT: (**a**) Follicle quality in HOCT cultured under static (PD) and dynamic (PB) conditions versus fresh tissue (D0). (**b-d**) Representative images of grade I (**b**), II (**c**) and III (**d**) follicles. (**e**) Follicle stages in HOCT cultured in static (PD) and dynamic (PB) conditions versus fresh tissue (D0); (**f–h**) Representative images of primordial (**f**), primary (**g**) and secondary (**h**) follicles. Bar = 20 μm. All the experiments were performed in triplicate (n = 3), and the values represent cumulative percentages. *P < 0.05 and **P < 0.01 versus D0; #P < 0.05 and ##P < 0.01 versus PD.

**Figure 7:**
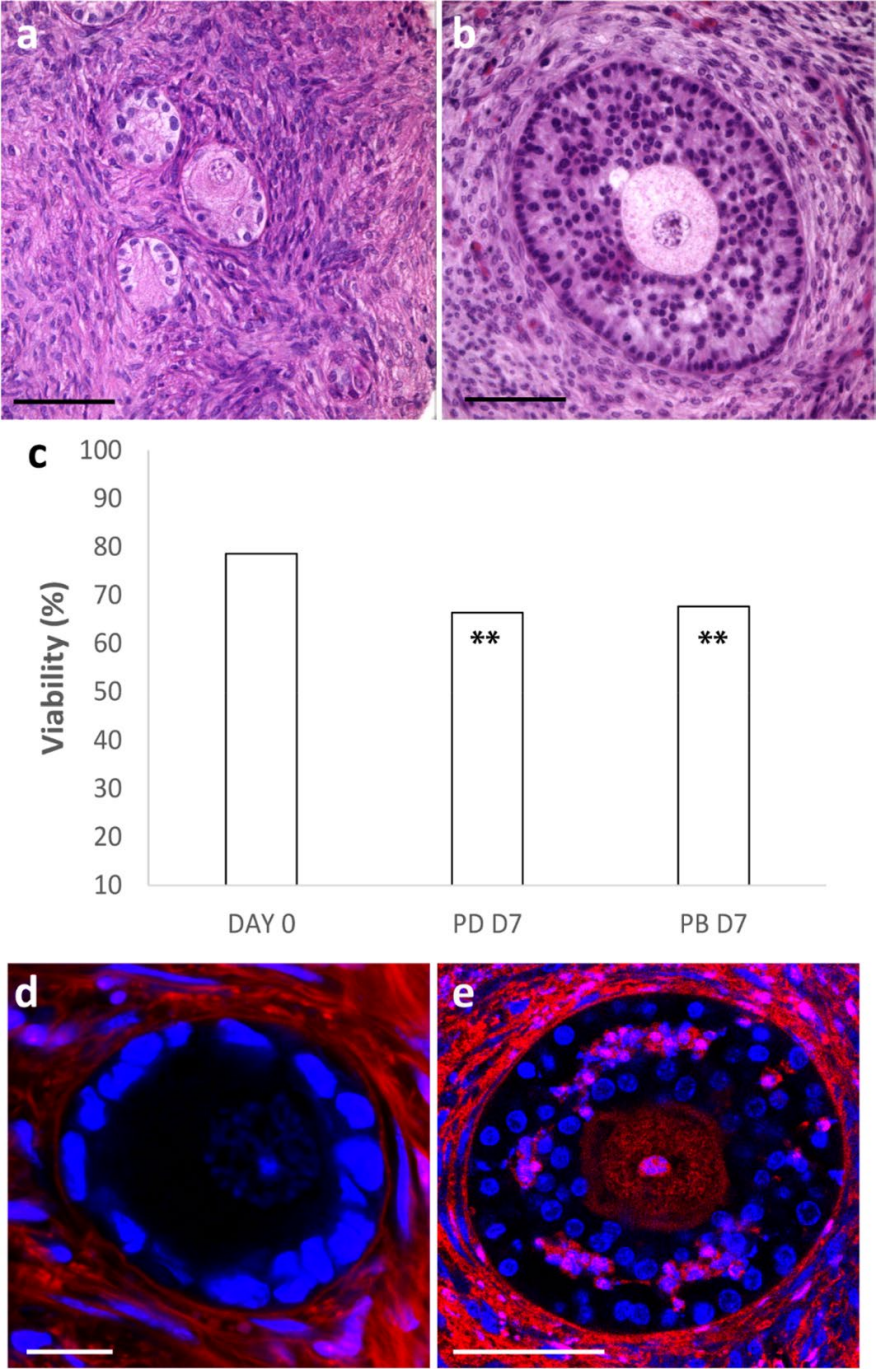
Histology and follicle viability in fresh and cultured HOCT: (**a**, **b**) Histology of HOCT cultured under (**a**) static (PD), and (**b**) dynamic (PB) culture conditions; Bar = 50 μm. (**c**) Follicle viability of HOCT cultured under static (PD) and dynamic conditions (PB) versus fresh tissue (D0). **P < 0.01 versus D0. (**d**, **e**) Representative confocal micrographs of: a live (**d**) and a dead (**e**) follicle. Blue, Hoechst 33342 ± stained nuclei; red, extracellular matrix and dead cells stained with live ± dead far-red; magenta, nuclei of dead cells. Bar = 20 μm.

Ovarian biopsies from three patients were collected and processed as described below. Unfortunately, HOCT was available in very limited amounts being a precious biological material. The results obtained with bovine tissue showed that static culture in PD was more effective than static culture in CD. For these reasons, the efficacy of culturing HOCT in the dynamic PB was compared only with PD, the best-performing static culture with bovine tissue. Histological characterization of fresh and cultured tissues (n = 3; total follicle number, 834: fresh tissue D0—444; D7—210, PB; 180, PD) showed that dynamic culture of HOCT strips in PB yielded significantly fewer grade III follicles than static culture in PD (P < 0.05; Fig. 6a), exhibiting a follicle grading (Fig. 6b–d) similar to fresh tissue at D0. Strips cultured in PB and PD showed a slight but significant decrease (P < 0.01) of grade I follicles compared to D0 (Fig. 6a). The percentage of secondary follicles in strips cultured in PB was significantly greater than (P < 0.01) in PD (Fig. 6e). A significant decrease of primordial follicles (P < 0.01) and a marked increase of primary and secondary follicles (Fig. 6f–h) was observed in cultured tissues compared to fresh tissue at D0. Representative histological images of tissue cultured in PD (Fig. 7a) and PB (Fig. 7b) showed healthy stromal cells distributed around the follicles. Analysis of follicle viability at the confocal microscope (n = 3; total follicle number, 779: fresh tissue D0—285; D7—259, PB; 235, PD) showed that a large number of follicles was viable under both culture conditions at D7 (Fig. 7c).

### Hormone secretion

Table 1 shows the concentration (mean±standard deviation) of 17β-estradiol (E2) and progesterone (P4) in spent media collected from PB and PD cultures of BOCT and HOCT at D7. The assessments (n = 3) were done on the same bovine and human samples evaluated for histology and viability (please see above). E2 concentration was consistently higher and P4 concentration consistently lower in PB than in PD culture medium. The E2 to P4 ratio for tissue dynamically cultured in PB was ~ 35 and 28 times higher than in PD for bovine and human tissue, respectively.

**Table 1:** Hormone secretion in cultured BOCT and HOCT.

	PB D7	PD D7
Bovine		
Estradiol (ng)	0.0038 ± 0.002	0.001 ± 0.0005*
Progesterone (ng)	2.35 ± 0.15	21.95 ± 1.5**
Ratio E2/P4	1.6 × 10^−3^	0.046 × 10^−3^
Human		
Estradiol (ng)	0.0051 ± 0.001	0.0015 ± 0.03*
Progesterone (ng)	2.03 ± 1.04	16.5 ± 4.17**
Ratio E2/P4	2.5 × 10^−3^	0.09 × 10^−3^

## Discussion

Growing follicles in vitro could be a promising future option with great potential in a wide range of reproductive techniques including fertility preservation, production of mature oocytes for autologous and heterologous in vitro fertilization, and oocyte banking. It could also provide an in vitro model system to advance knowledge of the intricate mechanisms that regulate follicle dormancy, growth and maturation in vivo^27^ and for drug testing^28,29^. To date, successful human in vitro folliculogenesis until the production of mature metaphase II oocytes has been achieved using the multi-step culture strategy^8,9^, but with low efficiency and some criticism on oocyte normality. Further optimization of microenvironmental culture conditions than currently done might help overcome such limitations and increase the efficiency of ovarian cortical tissue culture to generate a higher number of healthy secondary follicles. This represents a crucial starting point for the subsequent culture steps needed to achieve a complete follicular growth and production of mature oocytes for clinical purposes. Thus far, to better mimic the physiologic processes involved in follicular activation and growth, few studies have focused on the possibility to support in vitro folliculogenesis in dynamic culture systems that guarantee an efficient exchange of nutrients and metabolic products^30–32^. In this paper, we investigated the capacity of a novel bioreactor prototype to support in vitro the early stages of folliculogenesis. To this purpose, we compared the culture of BOCT and HOCT in the novel dynamic perifusion bioreactor with static culture in PD^17^. Since HOCT is precious and available in very limited amounts, in the first phase of this study experiments were performed on bovine prior to using human tissue. The bovine is adopted as an animal model for its similarities to human reproductive physiology in terms of size, structure and consistency of the ovary, length of folliculogenesis, presence of a dominant follicle, follicle size and for its easy availability at slaughterhouses^33–36^. For this reason, the effect of culture in the novel bioreactor on follicle growth, quality, viability and hormone secretion was evaluated first for BOCT in comparison to static culture in dishes, and only later on for HOCT. Histological analysis confirmed that culture in PD enhances follicle progression and quality with respect to CD^17^ and evidenced the benefits of dynamic culture. In fact, a significantly higher proportion of healthy follicles and secondary follicles were observed in dynamically cultured BOCT and HOCT as compared to static culture. Such data suggest that the environmental conditions generated by dynamic culture may allow to overcome some of the factors limiting follicle growth in the static conditions currently adopted in most procedures described in literature. In fact, in spite of the fact that several groups have tried to optimize media composition and the procedures for static culture of human and animal ovarian cortical strips, the percentage of healthy secondary follicles is currently still unsatisfactory^8,37^. The finding that dynamic culture promotes the growth of a higher number of healthy secondary follicles strengthens the hypothesis that the physical–chemical characteristics of the culture microenvironment, such as oxygen and nutrients supply, waste metabolites removal and the physical forces exerted on tissue and follicles, could play a crucial role in sustaining folliculogenesis in vitro. The model predictions, for the bioreactor geometry and operating conditions used for this study, indicate that the strips are well perifused with medium, and that the flow pattern around the strips effectively enhances oxygen availability inside the strips and the exchange of metabolically relevant dissolved solutes between medium bulk and tissue over culture in PD. The importance of oxygen availability for the activation of follicles and growth in vitro has been recently emphasized^17,38^. In the ovary, primordial and primary follicles are localized in an environment characterized by low O_2_ tension (hypoxic ovarian cortex), whereas secondary follicles are generally found in the vascularized medulla where O_2_ tension is higher^39^. Accordingly, increasing oxygen consumption rates have been reported during follicle transition from primordial to primary and to secondary stage^40^. Moreover, hypoxia has been shown to induce the dormancy of primordial follicles through the expression of Foxo3 in oocytes^40,41^ whereas an increased oxygen supply from blood vessels reportedly promotes the activation of dormant primordial follicles in mouse ovaries^42^. Within this framework, the increase of secondary follicles observed in PB is likely promoted by the increased oxygen availability deriving from good medium oxygenation and efficient oxygen transport from medium bulk to tissue.

Our PB model predictions indicate that medium perifusion is not only beneficial to the transport of metabolically relevant dissolved solutes (such as oxygen). In fact, the flow transport model predicts that the tissue strips cultured in PB are also subjected to fluid-mechanical shear stresses that, together with the application of solid compressive strains, are expected to contribute to eliciting a tissue biochemical response that is completely absent in both CD and PD static culture. Recent data^43^ suggest the existence of a complex interplay between biochemical and biomechanical signals in developmental processes, with feedback loops between the two types of signals^44^. Ovarian cells sense and transduce internally generated forces (e.g., elasticity, stiffness, etc.) or external forces (e.g., pressure, solid compressive or tensile forces, or fluid-mechanical forces) into nuclear processes, which may change gene expression^45^. Mechanical signalling can transmit information to other cells either directly via cell–cell junctions or indirectly via cell-ECM interactions^44^. As for the effect of solid mechanical strain, mechanical fragmentation, or enzymatic digestion, of the ovarian cortex has been shown to release tension on oocytes caused by the stiff ECM and the neighbouring granulosa cells; this activates the dormant follicles by disrupting the Hippo-pathway^46–48^ or by triggering FOXO3 nuclear translocation, respectively^49^, and promotes follicle progression and health^48^. The uniaxial compression of the soft ovarian tissue in the novel PB may be expected to cause an elongational and shear flow in the unconstrained directions (i.e., squeeze flow) that stretches follicle cells in these directions^50^. Biaxial stretching of densely packed quiescent epithelial cell monolayers has been shown to induce rapid cell cycle re-entry and progression to the S-phase by the consecutive nuclear accumulation and transcriptional activity of Yap1 and β-catenin^51^. Along the same line of thinking, Fletcher et al.^52^ have shown that stretching follicle cells of *Drosophila* causes cell flattening and the dilution of the Hippo pathway proteins that leads to the reduction of Hippo dimerisation and to the nuclear translocation of Yki (called YAP in mammalian cells) eventually activating cell proliferation. Consistent with that above, the application of a short tensile stretch has been reported to promote follicle progression and health in subsequently cultured ovarian tissue^53^. Additionally, model predictions show that flowing medium exerted on tissue fluid-mechanical shear stresses in the tens of mPa range. Shear stresses in such a range have been shown to stimulate in cell-specific fashion the biochemical response of a number of cells in 3D culture^54–59^. On these grounds, it may be speculated that tissue mechanical stimulation, resulting from the combination of solid compressive strains and fluid-mechanical shear stresses likely contributed to the enhancement of healthy secondary follicle growth during culture in the novel dynamic PB. However, elucidation of the mechanisms through which enhanced oxygen availability and solutes exchange with tissue coupled to mechanical stimulation improved the outcome of dynamic ovarian tissue culture in our novel PB to the extent herein reported requires further investigation.

A good culture microenvironment, apart from maintaining the follicles, should also keep stromal cells healthy long-term to enable a physiological cross-talk between stromal and follicular compartments. Our previously developed live-dead confocal assay is a valuable procedure for the characterization of follicle viability, but it allows the evaluation of stromal (and follicular) cell viability only at a limited depth (100–150 μm) inside the tissue strip^60^. To overcome such limit, the viability of the whole tissue was characterized by assaying LDH activity in the spent media from BOCT culture under static and dynamic conditions. The health of the whole tissue was significantly better at all analysed time points for ovarian cortical tissue dynamically than statically cultured. Consistent with the histological analysis, this suggests a better maintenance of stromal-follicular interaction in dynamic culture^61^. The level of steroid hormones in the spent medium during ovarian tissue culture is another relevant endpoint of follicle growth and function in vitro. In fact, E2 concentration is directly proportional to the number and health of granulosa cells, and consequently to the various follicle stages^62^. In this study, the increased levels of E2 detected in medium collected from BOCT and HOCT cultures in PB in comparison to PD static culture, are likely correlated to the observed higher proportion of healthy secondary follicles, consistent with that reported in literature^63^. The higher P4 concentrations and the lower E2/P4 concentration ratio in BOCT and HOCT in medium from PD versus PB culture demonstrate that dynamic culture in the current bioreactor design promotes a better maintenance of follicle physiology than static culture in PD. Such an outcome is in good agreement with previous studies suggesting that increased progesterone levels in spent media of HOCT culture are related to a premature luteinization of growing follicles ^30,64–67^.

## Conclusions

In this paper, ovarian cortical strips were cultured in simple medium without Follicle Stimulating Hormone or other supplements to more clearly assess the effect of our dynamic bioreactor design versus static culture in conventional dishes on follicle growth, health and hormone secretions. Taken together, the findings indicate that culture in dynamic bioreactors could open new interesting and promising perspectives to increase the efficacy of a multi-step culture strategy. This could lead to the enhancement of both quality and percentage of secondary follicles at the end of the first step of culture, which is fundamental pre-requisite for a successful in vitro folliculogenesis.

## Materials and methods

The materials and methods used for this study are similar to those used for prior studies published by our group^17,25^ and occasionally reproduce them *verbatim* in the following.

### Study design

The study was structured in two experimental phases. In the first phase, we investigated the effects of 7 days dynamic culture of strips of fresh bovine ovarian cortical tissue (BOCT) in the novel PB on follicle growth, health and hormone secretion. Tissue from each of three ovaries (n = 3) was cultured in vitro in parallel in PB and under static conditions in CD or PD, and the static cultures were used as controls. The histological assessment of follicle stages quality, and viability was performed by conventional and confocal optical microscopy, respectively. Whole tissue viability and E2 and P4e secretion were characterized with spectrophotometric assays on spent media collected during and at the end of culture. In the second phase, we investigated the effects of dynamic culture in the PB on strips of fresh HOCT. Tissue from each of three ovaries (n = 3) was cultured in vitro in parallel in PB and under static conditions in PD, and the static culture was used as control. Follicle stages and quality, follicle viability and hormone secretion were assessed as described above. At the end of culture, 6 strips from each dish were treated for histology and 4 strips for viability assessments.

### Materials

Lumox culture dishes with gas-permeable bottom 50 mm in diameter (PD) were purchased from Sarstedt AG & Co. KG (Nuembrecht, Germany). Conventional dishes 50 mm in diameter were purchased from Falcon (Sigma-Aldrich, Milan, Italy). Leibovitz's L-15 medium, α-MEM Glutamax medium (code number 32571), insulin transferrin selenium (ITS) 100×, and live/dead fixable Far Red stain were purchased from Invitrogen (Milan, Italy). Penicillin streptomycin (Pen-Strep) 100×, amphotericin B 250 μg/ml, bovine serum albumin BSA, L-ascorbic acid, L-glutamine 200 mM, Hoechst 33342, fructose, α-thioglycerol, and eosin-Y were purchased from Sigma Aldrich (Milan, Italy). Mayers's hematoxylin and paraffin wax were from Carlo Erba (Milan, Italy). Estradiol and progesterone solid phase enzyme immunoassay kits were obtained from DRG (Marburg, Germany). LDH kit was obtained from Merck (Sigma-Aldrich, Milan, Italy). The tissue chopper was purchased from McIlwain, Mickle Laboratory Engineering Company Ltd (Surrey, UK). Pump tubing was purchased from Cole-Parmer (Milan, Italy) and the peristaltic pump from Ismatec (Enco, Venice, Italy).

### The custom tissue slicer

A tissue slicer was purposely designed to obtain slices of ovarian cortical tissue of more reproducible thickness and in less labour-intensive fashion than commercial slicers, and to be more easily handled. The slicer design is a modification of the commercial slicer by Thomas Scientific intended to improve on its characteristics. Supplementary Fig. 1a shows that the slicer consists of three parts and a cutting sleigh: (1) the lower slab, with an ellipsoidal cavity to maintain the ovary firm in place; (2) the intermediate slab, with an ellipsoidal through-hole to host the ovary and the handle to hold the slicer; (3) the upper slab with the guide for the cutting sleigh and a distancer for the blade (Supplementary Fig. 1b). The cutting sleigh (n.4) features four ellipsoidal cavities 500 μm deep. To assemble the slicer, slab 1 and 2 are held together with a couple of screws and springs. An additional couple of screws and springs is used to keep slab 2 and 3 together and to exert a steady pressure on the ovary that is being cut. A slicer prototype was manufactured by subtractive manufacturing technique from solid polymethylmethacrylate (PMMA), a clear see-through polymer, easy to clean and sterilize. The reproducibility of the strip thickness was assessed by using the slicer to cut tissue slices out of fresh bovine ovaries. The strips thickness was characterized on stereomicroscopic images by means of the Image J software (NIH, Bethesda, MD, USA). 10 images of each strip (vertically oriented) (Supplementary Fig. 1c, asterisk) were acquired and at least 3 different fields were randomly examined with the line-selection tool of Image J software. The average strips thickness was 588 ± 56 μm (Supplementary Fig. 1d).

### The dynamic perifusion bioreactor

The dynamic perifusion bioreactor (PB) used for this study is shown schematically in Fig. 1a. Its development and structure are described in detail elsewhere^25^. Briefly, the PB consists of two conical halves, held together with screws, making up a 6.9 ml culture chamber. A silicon O-ring prevents liquid leakages. A porous support is located at the top of the lower bioreactor half and separates the inlet from the outlet half. Strips of ovarian cortical tissue are laid on the porous support enveloped between two polyester nets, that hold them in fixed positions in space, and are continuously perifused with medium mainly flowing perpendicular to the porous support. Ribs protrude from the vaults of both bioreactor halves with the function of guiding medium flow and of exerting compressive strains on the tissue strips (those in the upper bioreactor half). Bioreactor prototypes for this study were manufactured in our workshop by adapting to the bioreactor specifications commercial devices made of food-grade polypropylene designed for other applications.

### Collection and preparation of ovarian tissue strips

Bovine ovaries (n = 3) were collected from slaughtered animals (8–24 month) at the time of evisceration (Slaughterhouse Straccione, San Marcellino, Caserta, Italy; CEE accreditation number 1403/M) and were transported within 2 h to the lab in Leibovitz's L-15, 1% Pen-Strep, 1 μg/ml amphotericin-B, at 4 °C.

Human ovarian biopsies were collected from three women (age = 25.6 ± 4.8 years) during laparoscopic surgery for benign gynaecologic conditions after obtaining written informed consent, and were transported to the lab as described above. The use of human tissue was approved by the Ethics Committee of Regione Campania (ASL NA1 Centro, Naples, Italy; reference number 57/CE 15th February 2017) in accordance with the relevant guidelines and regulations. This study was conducted in accordance with the principles of the Declaration of Helsinki. Cortical tissue slices were obtained from bovine ovaries (n = 3) or human ovarian biopsies (n = 3) with the custom slicer, avoiding areas with visible antral follicles. Slices were placed in handling medium (Leibovitz’s L-15, 2 mM glutamine, 3 mg/ml BSA, 1% Pen-Strep, 1 μg/ml amphotericin B), cut into 1 mm × 1 mm strips with a tissue chopper, washed twice in fresh handling medium, and randomly allocated in group of 10 to each culture condition.

### Ovarian tissue culture

Ovarian cortical strips were cultured in α-MEM culture medium supplemented with 3 mM glutamine, 0.1% BSA, 1% Pen-Strep, 1% ITS (10 μg/ml Insulin, 5.5 μg/ml Transferrin, 0.67 ng/ml Selenium), 1 μg/ml amphotericin-B, 50 μg/ml ascorbic acid. Hereinafter such supplemented medium is simply referred to as medium.

### Dynamic culture in perifusion bioreactors

#### Bioreactor operation

The set-up used for the dynamic culture experiments is schematically shown in Fig. 1b. Briefly, medium was continuously circulated at 4 ml/min with a peristaltic pump from the medium reservoir to gas-permeable tubing (to enrich medium with dissolved O_2_ and to remove excess dissolved CO_2_), from there to the PB bioreactor, and eventually back to the reservoir.

For the culture experiments, 10 tissue strips (1 × 1 mm, 588 ± 56 μm thick) were positioned on three rows in a 3-4-3 staggered pattern at a nominal 1 mm distance from one another at the centre of the porous support in the bioreactor outlet half, and were held in fixed space positions by sandwiching them between two multifilament woven medical-grade polyester nets (330 × 290 μm mesh size). The compound mechanical challenge exerted by the ribs in the upper bioreactor half and the nets enveloping tissue yielded a solid compressive strain ranging from −3 to −13% with respect to the strip pre-compression thickness on the tissue strips that were used for this study, as characterized by 3D profilometry. Bioreactor geometry and operation was designed to yield good tissue perifusion (medium flow around the strips) and suitable fluid-dynamic stimulation (effected by the shear stresses acting on tissue) with three-dimensional (3D) mathematical models of flow transport in the bioreactor, as described below.

#### Mathematical modelling

Medium flow in the bioreactor voids was described according to the Navier–Stokes equations^26^ for an incompressible liquid with viscosity and density equal to water at the operating temperature. Flow through the nets was described in terms of the Darcy–Brinkman equation for an isotropic porous slab with uniform Darcy permeability^68^. The following boundary conditions completed the description of flow transport: no-slip at solid surfaces (i.e., bioreactor inner walls and porous support, tissue); solid surfaces impermeable to medium; atmospheric pressure at bioreactor outlet; set medium feed flow rate at bioreactor inlet; continuity of velocity and flux at compartment interfaces. Oxygen transport in medium in the bioreactor was described according to the diffusion–advection equations^26^. Oxygen transport and metabolic consumption inside tissue was described according to the diffusion–reaction equation assuming pseudo-homogeneous tissue and Michaelian oxygen metabolic consumption kinetics. The following boundary conditions were applied: solid surfaces (i.e., bioreactor inner walls and porous support) impermeable to oxygen; set dissolved oxygen concentration in feed stream; continuity of oxygen concentrations and concentration gradients at compartment interfaces. Model predictions were obtained for the following parameter values^69^: dissolved oxygen concentration in feed stream, C_B_ = 0.216 mol/m^3^ (corresponding to a pC_O2,gas_ of about 21%); effective oxygen diffusivity in tissue, D_O2,eff_ = 2.8 × 10^−9^ m/s^2^; oxygen diffusivity in medium, D_O2,m_ = 3.5 × 10^−9^ m/s^2^; maximal oxygen consumption rate by tissue, V_max,O2_ = 2 × 10^−2^ mol/(s m^3^_tissue_); Michaelis constant for oxygen consumption, K_m,O2_ = 5 × 10^−3^ mol/m^3^. Bioreactor materials were assumed impermeable to oxygen.

The resulting flow and oxygen mass balance equations were solved numerically with the commercial FEM code Comsol Multiphysics ver. 5.3 (Burlington, MA, USA) with a 10^−6^ convergence percent error.

#### Sterilization and tissue loading

Prior to loading the tissue strips in the bioreactor, the whole experimental set-up was assembled and sterilized by circulating 4 ml/min of 2% (v/v) glutaraldehyde solution in sterile water for 20 min. Then, the circulation loop including the PB was rinsed twice with sterile water for 20 min to remove glutaraldehyde residues, washed with PBS supplemented with 25 μg/ml amphotericin B and 1% Pen-Strep for 20 min, and rinsed again with sterile water for 10 min. After that, the circulation loop and PB was primed by flushing a pre-heated medium (α-MEM supplemented with 0.1% BSA, 1% Pen-Strep, and 1 μg/ml amphotericin-B) and the PB was carefully de-aerated with an upside-down inversion and gentle shaking. Then, the whole set-up was incubated at 37 °C in a humidified atmosphere with 5% CO_2_ in air for 30 min. Subsequently, the PB was disconnected, emptied, and 10 strips of ovarian cortical tissue from any given ovary, prepared as described above, were randomly selected among those available, and positioned between the two polyester nets as reported above. Then, the whole set-up was filled with medium, de-aerated again, as described above, incubated at 37 °C in 5% CO_2_ and 95% humidity air atmosphere, and tissue was cultured for 7 days. Half volume of spent medium was replaced with fresh medium every 48 h.

### Static culture in dishes

The O_2_ transport model for each static bioreactor was based on previously published models for dissolved O_2_ transport in ovarian cortical tissue cultured under a layer of stagnant medium in CDs and PDs^17^. The same assumptions and parameter values were used for the O_2_ transport models in tissue cultured in static CDs and PDs, and for the associated simulations. In CDs, the bottom dish surface was assumed impermeable to oxygen. In PDs, the dissolved oxygen concentration in the liquid layer below the strips was assumed in equilibrium with the gas in the incubator. Tissue strips were cultured in CDs similarly to PBs. 10 strips of ovarian cortical tissue from any given ovary were randomly selected, positioned at CD or PD bottom, and cultured in 5 ml medium. Tissue was cultured under the same conditions as the PBs, and half volume of spent medium was replaced with fresh medium every 48 h.

At the end of any culture, 5 strips from every bioreactor were prepared for histology and 5 strips for viability assessment. Spent medium was collected every 48 h before medium exchange.

### Histology

For histological analysis, the cortical strips were fixed in Bouin's solution, dehydrated in increasing serial ethanol concentrations, embedded in paraffin, and 5 μm serial sections were stained with hematoxylin and eosin. For each experiment and culture conditions, six strips were serially sectioned and entirely examined and the follicles grading and staging were assessed by two blinded expert observers: only follicles in which the germinal vesicle was well visible were classified. Follicle quality was graded as previously described^70^. Briefly: grade 1 follicles were spherical with homogeneously distributed granulosa cells (GCs) and oocyte presenting a homogenous cytoplasm and slightly granular nucleus, with well visible spherical condensed chromatin; grade 2 follicles had GCs non-uniformly distributed around the spherical oocyte; grade 3 follicles had pyknotic GCs and distorted and/or vacuolized oocyte. Follicle staging was scored according to Gougeon's criteria^7^, as follows: primordial follicles have a single layer of flat GCs; primary follicles have a complete single layer of cuboidal GCs; secondary follicles have two or more complete layers of cuboidal GCs.

### Viability assessment

#### Confocal laser microscopy

For the viability assessment, the strips were incubated under shaking for 3 h at 4 °C in Dulbecco's PBS with 1 μg/ml Live/Dead fixable far red stain and 10 μg/ml Hoechst 33342, fixed in 4% paraformaldehyde in PBS for 2 h at RT, washed in fresh PBS and incubated at 4 °C overnight in PBS plus 10 μg/ml Hoechst 33342^60^. The live/dead probe is resistant to fixation, reacts with free amines both in the cell interior and on the cell surface, and is excluded by cells with intact membranes. The strips were then optically cleared using the See DB clearing protocol^71^. Briefly, samples were serially incubated in 5 mL of 20%, 40%, and 60% (wt/vol) fructose, each for 6 h, 80%, 100% and 115% fructose (wt/vol) each for 12 h, with gentle agitation at RT. All fructose solutions were supplemented with 0.5% α-thioglycerol. To avoid compression, the strips were mounted in 115% fructose on a glass slide with 3 spacer coverslips (0.17 mm) placed on each side and covered with a coverslip. The samples were analyzed with a Leica TCS SP5 confocal scanning laser microscope (Leica Microsystems, Wetzlar, Germany) using a 405-nm diode laser for visualizing the nuclear label (Hoechst 33342) and a 633-nm helium–neon laser for the live/dead probe. Each strip was traversed using the z-position control, and fields to a depth of 300 μm from the tissue surface were imaged using a 63 × glycerol immersion objective. Follicles stained by far red have a dead oocyte, and were not considered viable. For each experiment and culture conditions, four strips were entirely examined by two blinded operators.

#### LDH analysis

The whole tissue viability of bovine ovarian cortex cultured in dynamic PB versus static PD and CD was characterized by assaying the concentration of LDH released in spent medium with a commercial kit according to the manufacturer’s instructions (Sigma-Aldrich, Milano, Italy). Briefly, spent culture medium from PD and PB cultures was collected, centrifuged at 200* g* for 10 min to remove insoluble particles, and 50μL of supernatant was added to each well of 96-well plates together with positive controls or NADH standards at different concentrations. After 2–5 min, the absorbance at 450 nm was measured with a microplate reader (Synergy HTX Multi-Mode Reader, Agilent, Milano, Italy) at the initial test time, t_initial_. Subsequent measurements were taken every 5 min. The last measurement, A450_final_, recorded was the penultimate measurement, or the value before the most active sample reaches or exceeds the end of the linear range (t_final_). The time of the penultimate measurement is t_final_. LDH activity at each time point was evaluated according to the following Eq. (1)

1 $$\mathrm{LDH}=\frac{B \times \mathrm{dilution\, factor}}{\mathrm{Reaction\, time} \times V}$$

where B is the amount (nmole) of NADH generated between t_initial_ and t_final_, the reaction time indicates the difference between Time final and initial, and V is the sample volume. LDH was expressed as nmole/min/mL (that is mUnit/mL). Measurements were performed in duplicate.

### Hormone assay

Estradiol (E_2_) and Progesterone (P_4_) solid-phase enzyme-linked immunosorbent assay kits were used for the quantitative determination of both hormones’ concentration in spent culture medium. Briefly, 500 µl of spent medium was collected from bovine and human tissue culture in PB and PD at the end of culture and stored at −20 °C until assayed. For the measurement of hormones concentration, the samples were thawed, allowed to warm to RT, and were assayed along that required by the kit manufacturer. 25 µl of medium samples and standards were dispensed in a 96-well plate. After addition of enzymes and reactants, and proper washing and incubation steps, the enzymatic reaction was stopped, and the absorbance of each sample was determined at 450 ± 10 nm with a microtiter plate reader. Each hormone concentration was determined by comparison to a standard calibration curve. Measurements were performed in duplicate.

### Statistical analysis

For follicles quality, staging and viability, the data are reported as cumulative percentages. Statistical analysis was performed according to Fisher's exact test for pairwise comparisons when overall significance was detected. LDH and Elisa assays were analysed by one-way ANOVA followed by the Tukey’s post-test with GraphPad Prism 9 software. Statistical significance was set at a value of P < 0.05.

## Supplementary Information

Supplementary Information.

## Data Availability

The data that support the findings of this study are available on request from the corresponding author, R.T.
