# Supplementary material for: Dynamic in vitro culture of bovine and human ovarian tissue enhances follicle progression and health
Source: Sci Rep. 2023 Jul 21;13:11773. doi: 10.1038/s41598-023-37086-0 (PMC10361967; doi:10.1038/s41598-023-37086-0)
Supplement: Supplementary file 1 — Supplementary Information. [file 41598_2023_37086_MOESM1_ESM.docx]

**Dynamic in vitro culture of bovine and human ovarian tissue**

**enhances follicle progression and health**

Vincenza Barbato^1,*^, Vincenzo Genovese^1,6,*^, Vincenza De Gregorio^1,*^, Maddalena Di Nardo^1§^, Angela Travaglione^1^, Luigi De Napoli^2^, Gionata Fragomeni^3^, Elisabetta M. Zanetti^4^, Satish Kumar Adiga^5^, Giuseppe Mondrone^6^, Thomas D'Hooghe^7,8^, Wengijng Zheng^7^, Salvatore Longobardi^7^, Gerardo Catapano^2^, Roberto Gualtieri^1^, Riccardo Talevi^1#^

^1^Department of Biology, University of Naples “Federico II”, Complesso Universitario di Monte S. Angelo, Via Cinthia, 80126 Naples, Italy

^2^Department of Mechanical, Energy and Management Engineering, University of Calabria, Via P. Bucci, 87030, Rende CS, Italy

^3^Department of Medical and Surgical Sciences, Magna Graecia University, Viale Europa – Loc. Germaneto, 88100 Catanzaro, Italy

^4^Department of Engineering, University of Perugia, 06125 Perugia, Italy

^5^Centre of Excellence in Clinical Embryology, Department of Reproductive Science, Kasturba Medical College, Manipal Academy of Higher Education, Manipal 576 104, India

^6^IVF Research, Education, Development S.r.l., via Josemaria Escrivà, 68, 81100, Caserta, Italy

^7^Global Medical Unit Fertility, Merck Healthcare KGaA, Frankfurter Strasse 250, 64293 Darmstadt, Germany

^8^Department of Development and Regeneration, Group Biomedical Sciences, KU Leuven (Leuven University), Gasthuisberg Campus, Herestraat 49, 3000 Leuven, Belgium

^#^Correspondence should be addressed to R.T. (email: riccardo.talevi@unina.it)

^§^Current affiliation: Institute for Biomedical Technologies ITB, National Research Council CNR, Via Moruzzi, 1 56124, Pisa, Italy

* V.B., V.G. and V.D.G. equally contributed to the manuscript and share first authorship

^#^Correspondence and requests of material should be addressed to R.T. (email: riccardo.talevi@unina.it)

^§^Current affiliation: ^2^Institute for Biomedical Technologies ITB, National Research Council CNR, Via Moruzzi, 1 56124, Pisa, Italy

**Supplementary Materials and Methods**

**Supplementary Figure S1**


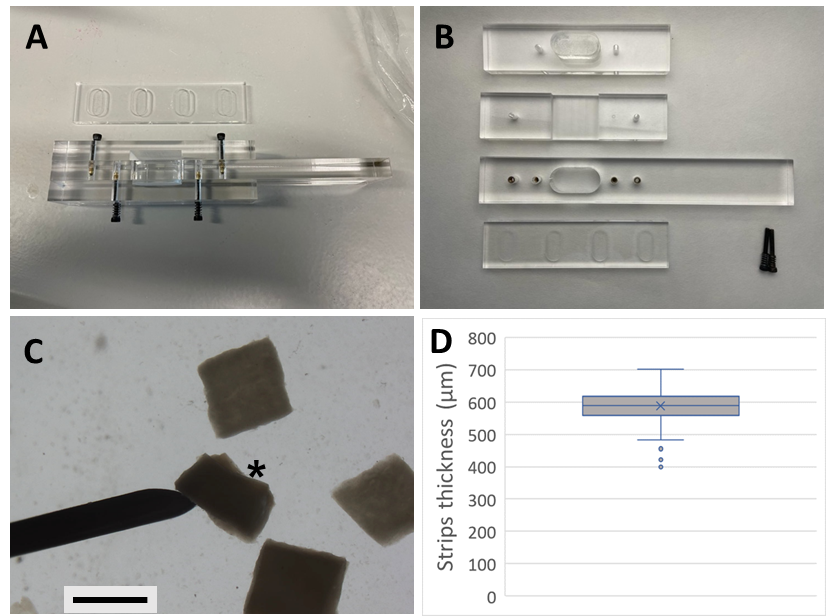


**Supplemental Figure S1. The custom-made tissue slicer**

a) the slicer fully assembled; b) the slicer components: 1. the lower slab, with an ellipsoidal cavity to maintain the ovary firm in place; 2. the intermediate slab, with an ellipsoidal through-hole to host the ovary and the handle to hold the slicer; 3. the upper slab with the guide for the cutting sleigh and a distancer for the blade; 4. the cutting sleigh featuring four ellipsoidal cavities 500 μm deep; c) exemplary slices of ovarian tissue (oriented slide, asterisk); d) thickness of the tissue slices (please see text for methods).
